# Self-sustained enzymatic cascade for the production of 2,5-furandicarboxylic acid from 5-methoxymethylfurfural

**DOI:** 10.1186/s13068-018-1091-2

**Published:** 2018-04-02

**Authors:** Juan Carro, Elena Fernández-Fueyo, Carmen Fernández-Alonso, Javier Cañada, René Ullrich, Martin Hofrichter, Miguel Alcalde, Patricia Ferreira, Angel T. Martínez

**Affiliations:** 1 Centro de Investigaciones Biológicas, CSIC, Ramiro de Maeztu 9, E-28040 Madrid, Spain; 2 Department of Bio- and Environmental Sciences, International Institute Zittau - Technische Universität Dresden, Markt 23, 02763 Zittau, Germany; 3 Department of Biocatalysis, Institute of Catalysis, CSIC, Marie Curie 2, E-28049 Madrid, Spain; 4 Department of Biochemistry and Molecular and Cellular Biology and BIFI, University of Zaragoza, E-50009 Saragossa, Spain

**Keywords:** 2,5-Furandicarboxylic acid, 5-Methoxymethyl furfural, Enzyme cascade, Biocatalysis, Oxidase, Peroxygenase, Renewable polyesters

## Abstract

**Background:**

2,5-Furandicarboxylic acid is a renewable building block for the production of polyfurandicarboxylates, which are biodegradable polyesters expected to substitute their classical counterparts derived from fossil resources. It may be produced from bio-based 5-hydroxymethylfurfural or 5-methoxymethylfurfural, both obtained by the acidic dehydration of biomass-derived fructose. 5-Methoxymethylfurfural, which is produced in the presence of methanol, generates less by-products and exhibits better storage stability than 5-hydroxymethylfurfural being, therefore, the industrial substrate of choice.

**Results:**

In this work, an enzymatic cascade involving three fungal oxidoreductases has been developed for the production of 2,5-furandicarboxylic acid from 5-methoxymethylfurfural. Aryl-alcohol oxidase and unspecific peroxygenase act on 5-methoxymethylfurfural and its partially oxidized derivatives yielding 2,5-furandicarboxylic acid, as well as methanol as a by-product. Methanol oxidase takes advantage of the methanol released for in situ producing H_2_O_2_ that, along with that produced by aryl-alcohol oxidase, fuels the peroxygenase reactions. In this way, the enzymatic cascade proceeds independently, with the only input of atmospheric O_2_, to attain a 70% conversion of initial 5-methoxymethylfurfural. The addition of some exogenous methanol to the reaction further improves the yield to attain an almost complete conversion of 5-methoxymethylfurfural into 2,5-furandicarboxylic acid.

**Conclusions:**

The synergistic action of aryl-alcohol oxidase and unspecific peroxygenase in the presence of 5-methoxymethylfurfural and O_2_ is sufficient for the production of 2,5-furandicarboxylic acid. The addition of methanol oxidase to the enzymatic cascade increases the 2,5-furandicarboxylic acid yields by oxidizing a reaction by-product to fuel the peroxygenase reactions.

## Background

Fossil resources are finite and the need for substituting petroleum-based materials with renewable materials is increasing in recent years [1]. 2,5-Furandicarboxylic acid (FDCA) is nowadays regarded as a promising precursor for the production of renewable and biodegradable bioplastics. Polyester formed by the condensation of this building block with ethylene glycol, known as poly(ethylene-2,5-furandicarboxylate) (PEF), is expected to substitute for other polyesters produced from fossil fuels, thanks to their renewable origin and their mechanical and gas barrier properties, which are even better than those of conventional poly(ethylene terephthalate) (PET) [2, 3]. Therefore, it is expected that PEF will be able to compete with PET not only in economic but also in environmental terms since its production lowers the balance of green-house gases emissions [4]. The first report on PEF enzymatic hydrolysis, which permits the recycling of its monomers, has been brought to light recently [5].

FDCA can be obtained from precursors that are formed upon the acidic dehydration of fructose, directly obtained from plants (as monosaccharide, in sucrose disaccharide and in inulin-type polymers) or by isomerization of glucose from hydrolysis of disaccharides (e.g., sucrose) or polysaccharides (e.g., in lignocellulosic materials). These precursors are mainly 5-hydroxymethylfurfural (HMF) and more recently 5-methoxymethylfurfural (MMF). The latter is obtained when fructose is dehydrated in the presence of methanol or by HMF etherification [6–8]. MMF is more stable upon storage than HMF, and fructose dehydration in methanol yields less side-products than when it takes place in water for HMF production. Successful attempts have been made to obtain polyesters from MMF and its derivatives [9], and a joint venture between BASF and Avantium, Synvina (www.synvina.com), has been created for sustainable industrial production of PEF from stable MMF.

In the above context, several patents [10–12] present methods for the production of FDCA from MMF, but all of them use oxidation catalysts such as bromide, cobalt, or manganese, along with other metals. Moreover, they describe processes that take place at high temperatures (in the range of 100–220 °C) and pressures (3–15 bar). The advantage of enzymes, which work under mild conditions (in aqueous solution, at room temperature and under atmospheric pressure), for the production of FDCA has gained momentum and several reports on the enzymatic oxidation of HMF to FDCA are available [13–15]. Particularly, the use of the natural portfolio of oxidases and peroxygenases in synthetic chemistry is very timely. While the former can perform selective oxidations producing H_2_O_2_ from atmospheric O_2_, the latter can use the released H_2_O_2_ to complete the full oxidation of complex molecules like in the whole conversion of HMF to FDCA which comprises three sequential oxidation steps [13].

**Fig. 1:**
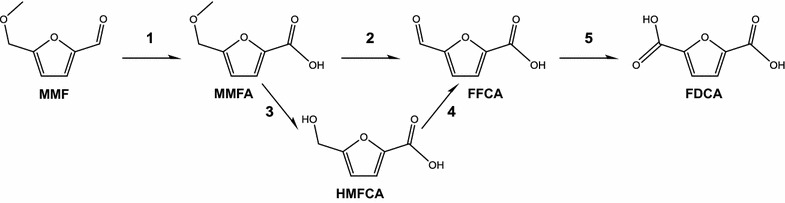
Scheme of the possible pathways for the oxidation of MMF into FDCA. MMF, 5-methoxymethylfurfural; MMFA, 5-methoxymethylfurancarboxylic acid; HMFCA, 5-hydroxymethyl-2-furancarboxylic acid; FFCA, 5-formylfurancarboxylic acid; and FDCA, 2,5-furandicarboxylic acid

MMF conversion into FDCA can involve three or four oxidation steps depending on whether the ether breakdown leaves an alcohol or a carbonyl function in the furfural molecule (Fig. 1 scheme, pathways 1/3–5 or 1/2/5, respectively). In the present study, a new self-sustained enzymatic cascade was developed for the production of FDCA from MMF—combining aryl-alcohol oxidase (AAO) [16], unspecific peroxygenase (UPO) [17], and methanol oxidase (MOX) [18]—identified the intermediate products by gas chromatography-mass spectrometry (GC–MS), estimated the conversion yields and established the oxidation pathway.

## Results

### Hydration of the carbonyl group in MMF

Comparison of ^1^H-NMR spectra in deuterated water and deuterated dimethylsulfoxide (DMSO-*d*6) allows detection of the aldehyde and the *geminal* diol signals to measure the degree of hydration at equilibrium. The MMF spectrum in DMSO-*d*6 showed six signals assigned to the aldehyde (9.6 ppm), the furanic ring (7.5 and 6.7 ppm), methylene ether (4.5 ppm), methyl (3.3 ppm), and residual DMSO (2.5 ppm) protons. On the contrary, the spectrum in sodium phosphate (pD 7.0) gave 8 signals assigned to the aldehyde proton (9.6 ppm) and its shifted counterpart hemiacetalic hydrated form (small signal at 6.8 ppm), the two ring protons (7.7 and 6.9 ppm) and their shifted counterparts (small signal at 6.5 ppm), the methylene ether (4.7 ppm) and methyl (3.5 ppm) protons, as well as the water protons (4.9 ppm). Integration of the aldehyde signal and its small shifted counterpart points towards a degree of MMF hydration ≤ 10%.

### MMF oxidation by AAO

**Fig. 2:**
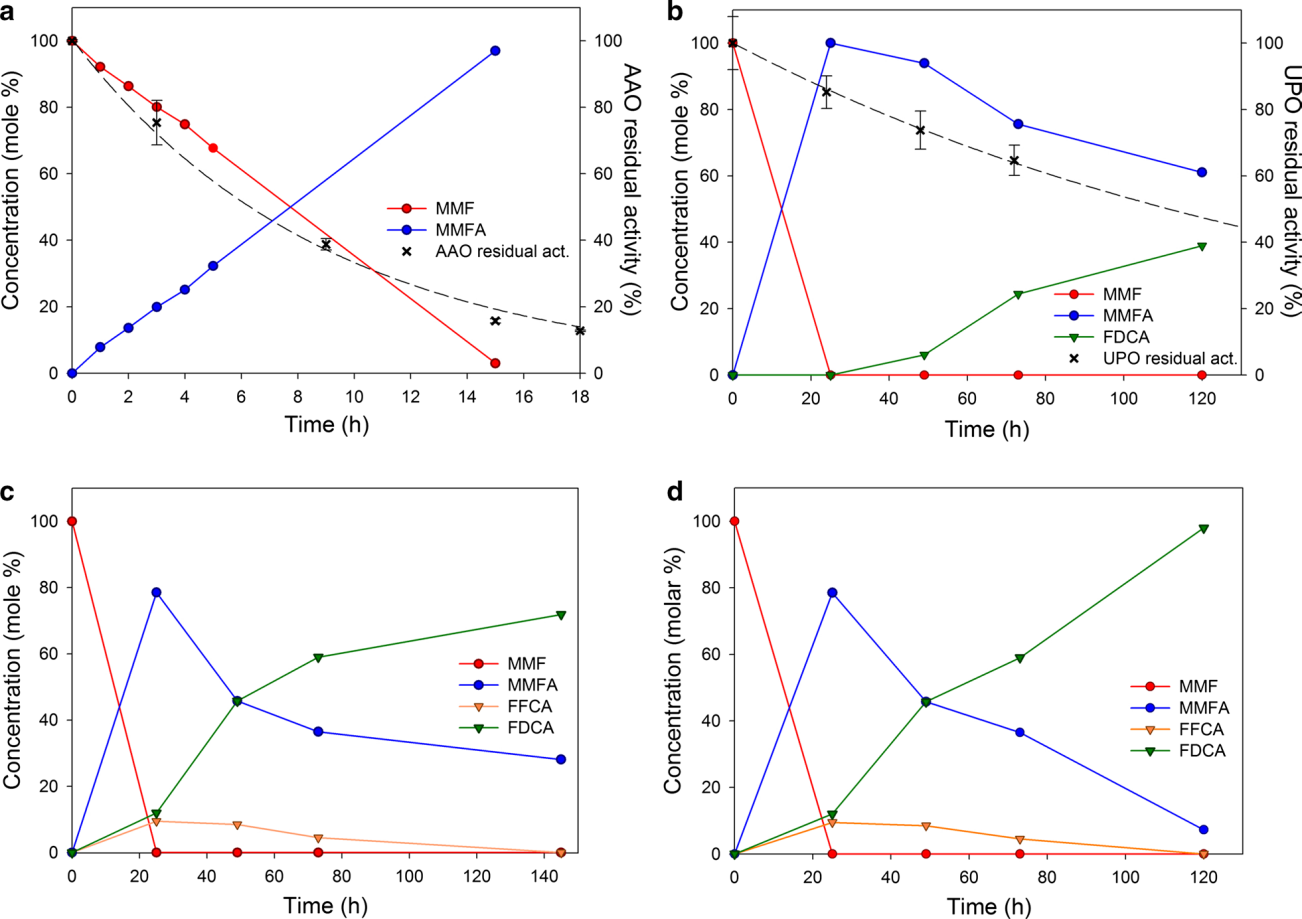
Time course of the reaction of MMF (1.5 mM) with: **a** AAO; **b** AAO, UPO and H_2_O_2_; **c** AAO, UPO and MOX; and **d** AAO, UPO, MOX and methanol (at 28 °C in 100 mM phosphate, pH 7). Enzyme concentrations were 5 µM (AAO and UPO) and 1 µM (MOX), while H_2_O_2_ and methanol final concentrations (in **b** and **d**, respectively) were 1.5 mM (added after 24, 48 and 72 h) and 1 mM (added after 72 and 96 h reaction). Dashed lines in **a** and **b** represent the AAO and UPO residual activities as a function of time, respectively. Compounds were identified and quantified by GC–MS, using the estimated response factors

AAO may oxidize aldehydes to acids if their carbonyl groups are partially hydrated to *gem*-diols [19]. To test the ability of AAO from *Pleurotus eryngii* to oxidize the *gem*-diol form of MMF, the compound was incubated with the enzyme and the reaction was analyzed by GC–MS. The reaction was completed after 15 h, using a substrate/enzyme ratio of 300 under the conditions described above (Fig. 2a). 5-(Methoxymethyl)-2-furancarboxylic acid (MMFA) accumulated over time and additional products were not detected, confirming that AAO does not show any activity on the resulting molecule. The above results show that the small hydration degree shown by NMR was enough for AAO oxidation of the MMF molecule to MMFA (step 1 in Fig. 1 scheme).

The kinetic constants for the AAO oxidation of MMF and related furfurals were indirectly measured as H_2_O_2_ release, by coupling the reaction of horseradish peroxidase (HRP) and a reagent that gives a colored product when peroxide is available (Table 1). Comparison with the kinetic constants estimated for related furfurals shows that the methoxyl moiety in MMF decreases the enzyme affinity (*K*_m_ is increased) and lowers reactivity (*k*_cat_ is slightly reduced). Both effects together result in much lower AAO catalytic efficiency for MMF than for the other two furfurals assayed.

**Table 1:** Catalytic constants for the oxidation of different furfurals by AAO

	*k*_cat_ (min^−1^)	*K*_m_ (mM)	*k*_cat_/*K*_m_ (min^−1^ mM^−1^)
MMF	15.8 ± 0.6	60.8 ± 5.5	0.35 ± 0.02
HMF	20.1 ± 0.6	1.6 ± 0.2	12.9 ± 1.2
DFF	31.4 ± 0.7	3.3 ± 0.2	9.4 ± 0.5

Reactions measured in 50 mM sodium phosphate (pH 7.0) at 25 °C. Means and standard deviations estimated from the fit to Michaelis–Menten equation. Kinetics were measured by triplicates

Residual activity estimations showed that AAO was active during the whole time of the reaction (dashed line in Fig. 2a) displaying a half-life of around 6 h that guaranteed the full conversion of MMF into MMFA. Catalytic performance parameters of AAO, including residual activity together with turnover number (TON), turnover frequency (TOF), and total turnover number (TTN) values under the described conditions, are provided in Table 2.

**Table 2:** Other catalytic parameters of AAO and UPO reactions (in Fig. 1a and b)

	Half-life (h)	TTN	TON	TOF (h^−1^)
AAO	6.3	8620	300	20
UPO	112.0	1400	594	5

TTN, TON and TOF were calculated using Eqs. 3–5, respectively (reaction times were 15 h for AAO and 120 h for UPO). Parameters estimated from single reactions using MMF (1.5 mM) as substrate and AAO and UPO (5 µM) as biocatalysts at pH 7.0 and 28 °C

### UPO reactions and AAO/UPO cascade

For the desired reaction to proceed further (from MMFA to FDCA), it was necessary to find a catalyst that could cleave the ether bond of the methoxyl group, to hydroxylate the 5-formylfurancarboxylic acid (FFCA) molecule and, if the ether cleavage left a hydroxyl group in the molecule, to oxidize the 5-hydroxymethyl-2-furancarboxylic acid (HMFCA) molecule to FFCA (steps 2/5 or 3–5, respectively, in Fig. 1 scheme). In this regard, the UPO from *Agrocybe aegerita* has been reported to cleave a variety of ether bonds [20], as well as to hydroxylate FFCA to FDCA in the presence of H_2_O_2_ [13].

To clarify the enzymatic pathway, UPO (5 µM) was incubated with MMFA (1.5 mM) in the presence of H_2_O_2_ (1.5 mM, final concentration). Detection of FFCA as the sole product (data not shown) revealed that UPO is indeed capable to cleave the ether bond of MMFA while forming an additional carbonyl group in the molecule. This suggests that the reaction mainly proceeds through step 2 (Fig. 1 scheme), rather than through steps 3 and 4, although traces of HMFCA were detected as well. Therefore, only steps 1, 2, and 5 in the Fig. 1 scheme would be required for the production of FDCA from MMF, which is advantageous, since it saves one catalytic step that would require additional H_2_O_2_.

**Fig. 3:**
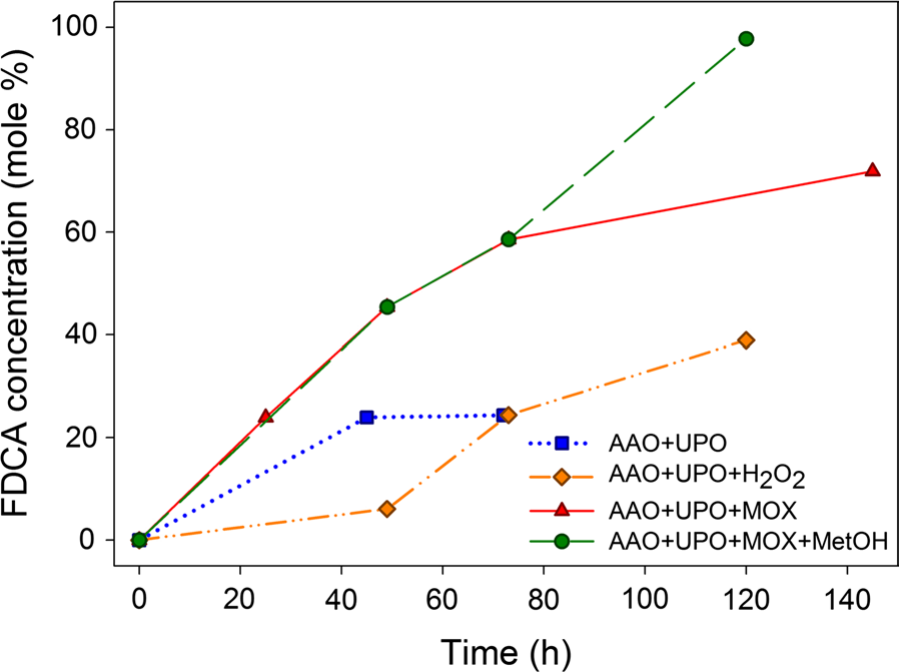
Comparison of FDCA production (as mole percentage of initial MMF) in: (i) AAO/UPO cascade (blue line); (ii) AAO/UPO cascade supplemented with added H_2_O_2_ (orange line); (iii) AAO/UPO/MOX cascade (red line); and (iv) AAO/UPO/MOX cascade supplemented which methanol (after 72 h of reaction). See Fig. 2 for enzyme activities and substrate concentrations

Given the two enzymatic activities described above—AAO’s ability to catalyze step 1 producing one equivalent of H_2_O_2_ and UPO being capable of catalyzing steps 2 and 5 consuming H_2_O_2_—an enzymatic cascade was assembled. AAO and UPO, both at a final concentration of 5 µM, reacted with 1.5 mM MMF (substrate/enzyme ratio of 300). This MMF concentration was selected to limit the UPO inhibition by H_2_O_2_ excess discussed below, although substrate saturation of AAO was not attained. Analysis of the reaction products revealed that, after 40 h, the reactions concluded—probably due to H_2_O_2_ depletion—resulting in approximately 25% conversion of initial MMF into FDCA (Fig. 3) together with 75% of MMFA (data not shown), which proved to be the main intermediate of the process and the bottleneck of the whole cascade.

### Addition of exogenous H_2_O_2_ to the cascade

To test if the limited FDCA yields of the AAO/UPO cascade were due to the depletion of H_2_O_2_, this stoichiometric UPO substrate was added at different times after 24 h of reaction (up to 1.5 mM) and the products were analyzed during the subsequent incubation. Analysis of the reaction products showed that the whole reaction was improved upon the addition of H_2_O_2_. The FDCA yield corresponded to 40% conversion of initial MMF and the remaining 60% accounted for MMFA, while only traces of FFCA were detected (Figs. 2b and 3). Although the concentration of H_2_O_2_ (exogenously added and in situ produced by AAO) may have been sufficient, the complete conversion of MMF into FDCA was not achieved.

UPO showed activity during the whole process, displaying robustness as a biocatalyst by maintaining high levels of residual activity throughout the reaction (Fig. 2b, dashed line). UPO half-life and other catalytic performance parameters (TON, TOF, and TTN) under the assayed conditions are shown in Table 2, together with those of AAO.

### Improvement of FDCA yield by MOX (and methanol) addition

The peroxygenase activity of UPO enables it to insert one O atom, which leads to ether breakdown, concomitantly with the formation of a carbonyl group in one of the products and a hydroxyl group in the other product [20]. As described above, the product of the reaction of MMFA with UPO was FFCA, in which a new carbonyl group was introduced. Consequently, UPO would release methanol as second fission product of the ‘quasi-benzylic’ peroxygenation reaction.

Therefore, with the aim of producing additional H_2_O_2_ to fuel the UPO reactions, commercially available MOX from *Pichia pastoris* was added to the enzymatic cascade described above to a final concentration of 1 µM (substrate/enzyme ratio of 1500). In this case, MOX catalyzes its canonical reaction, the oxidation of methanol to methanal and concomitantly, the reduction of O_2_ to H_2_O_2_. GC–MS analysis showed that upon the addition of MOX and the resulting increase of available H_2_O_2_, the UPO conversion was enhanced and FDCA yield reached 70% of the initial MMF concentration (Figs. 2c and 3). According to the analysis of products, the rate-limiting step of the UPO reactions (step 2 in Fig. 1 scheme) was demethoxylation, since MMFA was always the most abundant intermediate (up to 80% of initial MMF, after 24 h) compared to smaller amounts of FFCA (around 10% of the applied MMF).

To determine whether the improved FDCA yield (70%) was still limited by the amount of H_2_O_2_ available, exogenous methanol (1 mM final concentration) was added to the reaction after 72 and 96 h. In fact, with the addition of methanol (and subsequent H_2_O_2_ production), the FDCA formation further increased (Figs. 2d and 3), suggesting that the reaction was not limited by the activity of the biocatalyst. Thus, the FDCA conversion rose to 98% after 120 h, indicating that the limiting factor of the whole enzymatic cascade was the H_2_O_2_ availability. A summary of the conversion yields of MMF into MMFA, FFCA, and FDCA during operation (0–120 h) of the methanol-supplemented AAO/UPO/MOX cascade is provided in Table 3.

**Table 3:** Summary of the MMF (1.5 mM) conversion rates (mole %) to its three oxidized derivatives in the AAO/UPO/MOX cascade, supplemented with methanol (1 mM), at different reaction times

Time (h)	MMF	MMFA	FFCA	FDCA
0	100	0	0	0
25	0	66	10	24
49	0	46	9	45
73	0	35	6	59
120	0	2	0	98

Compounds were identified and quantified by GC–MS, using the estimated response factors

## Discussion

### AAO/UPO cascade for MMF oxidation

There is a wealth of oxidases involved in lignocellulose decay [21] that are potential biocatalysts for industry and are worth being studied [22]. In this work, the activity of *P. eryngii* AAO on lignocellulose-derived MMF is reported for the first time, which further widens/improves the application potential of AAO in FDCA production. In addition to fungal AAOs [13, 23, 24], other oxidases have proved to be suitable catalysts for the oxidation of HMF to FFCA or even FDCA employing O_2_ as co-substrate, as the so-called HMF oxidase (an intracellular bacterial enzyme from the same superfamily as AAO) [14, 15, 25].

MMF, which comes from the same renewable resources as HMF, appears as a better substrate for the synthesis of renewable polyesters, since it produces less dehydration by-products and displays higher stability upon storage. In the enzymatic oxidation of MMF, the drawback of AAO is its inability to produce the desired final product, FDCA. Acting alone, it lacks the ability to: (i) cleave the methoxy group in the MMF molecule and (ii) oxidize the carbonyl group in FFCA to FDCA. Nevertheless, the application of AAO in such process has the advantage that O_2_ is the only necessary reactant (co-substrate), apart from the chemical to be oxidized (substrate), to trigger the reaction, as it has been shown here. The production of H_2_O_2_ by AAO can be exploited for the creation of enzymatic cascades, in which another enzyme (peroxidase or peroxygenase) that uses it as electron acceptor (co-substrate) can be applied as downstream catalysts [13, 26]. Moreover, this is also a ‘smart’ way of destroying H_2_O_2_, which is an undesired by-product in terms of enzymatic performance and stability, forming H_2_O.

In the above context, the ability of UPO to perform an overwhelming number of different reactions [17, 27] allowed its application in the enzymatic synthesis of FDCA, not only from HMF [13] but also from MMF as shown in the present work. Here, it is shown that UPO is able to catalyze the cleavage of the ether bond in the MMF molecule thanks to the H_2_O_2_ produced by AAO. Furthermore, the fact that it catalyzed mainly the formation of a new carbonyl group in the molecule during ether fission (giving rise to FFCA from MMFA) saves one step in the enzymatic cascade designed, thereby reducing the need for H_2_O_2_ input. In general, the oxidation of MMF by AAO and UPO seems to proceed more efficiently than that of HMF [13, 28], due to the different polarity/reactivity of the methyl-ether functionality and the primary alcohol group, respectively.

Although the catalytic performances of the two biocatalysts are modest, with TTN values lower than 10^4^ and *k*_cat_ values lower than 1 s^−1^, the AAO/UPO cascade represents a good starting point for further improvement. The optimization of substrate and enzyme concentrations, required for an industrial exploitation of the cascade, would result in higher catalytic performance parameters.

The fact that UPO must catalyze two reactions (steps 2 and 5 in Fig. 1 scheme), whereas AAO does only one (step 1), causes a shortage in H_2_O_2,_ so that the desired reactions cannot be completed (Fig. 3, blue dotted line). To solve this limitation, a three-member enzymatic cascade was developed as discussed below.

### By-product oxidation to fuel the reaction

**Fig. 4:**
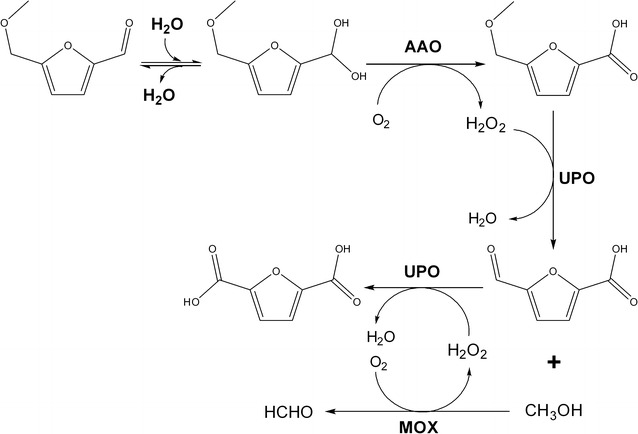
Scheme of the three-enzyme (AAO/UPO/MOX) self-sufficient enzymatic cascade developed for the production of FDCA from MMF

The addition of a third biocatalyst, MOX demonstrated that the limited FDCA yield (< 40% in the two-member AAO/UPO cascade) can be overcome by the in situ production of additional H_2_O_2_ to be used by UPO. Thus, a conversion of 70% from MMF to FDCA was attained using a three-enzyme cascade (AAO/UPO/MOX), which is fueled by the reduction of H_2_O_2_ (by UPO) and the oxidation of methanol (by MOX), two by-products generated by AAO and UPO, respectively. The respective results indicate that the 70% conversion is attained solely by the agents involved in the reaction as long as there is an atmospheric O_2_ input, as depicted in the three-member cascade scheme of Fig. 4.

The in situ self-generated fuel (H_2_O_2_ from the by-product methanol) here presented is a breakthrough in oxidative bioconversions. Other enzymatic cascades using UPO for oxygenations, in which methanol was applied as a sacrificial electron donor, have been reported, but the provenance of methanol was exogenous [26]. The production of methanal may be deleterious for enzymes if it reaches high concentrations, but such effect was not observed in the present study. The in situ generation of H_2_O_2_ has already been successfully applied in the bleaching industry using different flavoenzymes, such as glucose oxidase, alcohol oxidase [29] and cellobiose dehydrogenase/oxidase [30, 31].

Apart from improving the reaction yields, the cascade approach allows UPO to control the release of H_2_O_2_, since it is the enzyme itself that produces the methanol as substrate for MOX. It is well known that enzymes that bear heme groups, such as peroxidases and peroxygenases, are sensitive to high levels of their peroxide co-substrates resulting in deactivation via heme-bleaching. In fact, comparing the FDCA yields of the three-catalyst cascade with the cascade involving AAO and UPO to which H_2_O_2_ was added clearly indicates that UPO reactions proceed better if H_2_O_2_ is gradually supplied in situ by enzyme action (see red vs orange lines in Fig. 3). The addition of exogenous H_2_O_2_ provokes a sharp rise of its concentration that may lead to UPO inactivation. This results in a decrease of the enzyme’s half-life, thus lowering TTN, which negatively affects the degree of conversion into FDCA. Although complete MMF conversion was not achieved using the AAO/UPO/MOX cascade, the addition of a small amount of ‘extra methanol’ to the reaction mixture resulted in almost complete conversion (98%) of MMF into FDCA (Fig. 3). In fact, an excess of H_2_O_2_ (above the stoichiometric quantity required for the peroxygenation reaction) was necessary to overcome the reported catalase side-activity of UPO [32], which may consume some of the H_2_O_2_ by producing H_2_O and O_2_.

## Conclusions

In this work, a completely enzymatic approach to produce FDCA from MMF is reported for the first time. The synergistic activities of AAO and UPO catalyze such conversion through an enzymatic cascade involving the two biocatalysts and O_2_ to trigger the reaction. UPO benefits from the H_2_O_2_ released by AAO to yield modest amounts of FDCA with H_2_O and methanol as by-products. The addition of MOX, oxidizing methanol under release of additional H_2_O_2_, critically improved the FDCA yield that was before limited by the amount of H_2_O_2_ produced by AAO. Thus, 70% conversion was achieved with the only involvement of the three biocatalysts, O_2_ and the by-products of the reaction, as illustrated in the Fig. 4 scheme. This yield could be further enhanced to 98% by the addition of some exogenous methanol resulting in additional H_2_O_2_ for UPO.

## Methods

### Reagents

*P. pastoris* MOX (EC 1.1.3.13), *t*-butyl-methyl-ether, MMFA, FDCA, N,O-bis(trimethylsilyl)-trifluoroacetamide (BSTFA) and ^2^H_2_O were purchased from Sigma-Aldrich (Saint Louis, MO, USA). MMF (= 5-[methoxymethyl]-2-furancarboxaldehyde) was bought from AK Scientific, Inc (Union City, CA, USA). FFCA was purchased from TCI America (Portland, OR, USA). AmplexRed^®^ and HRP were obtained from Invitrogen (Walthem, MA, USA). H_2_O_2_ and DMSO-*d*6 were from Merck (Darmstadt, Hessen, Germany).

### Enzyme production

AAO (EC 1.1.3.7) from the fungus *P. eryngii* was heterologously obtained from recombinant *Escherichia coli* W3110 harboring the pFLAG1 vector with the mature AAO cDNA (GenBank accession number AF064069). The enzyme was produced as inclusion bodies and further in vitro activated and purified as previously described [33].

PaDa-I variant of *A. aegerita* UPO (EC 1.11.2.1) was produced in *P. pastoris*, harboring the pPICZ-B-PaDa-I vector, grown in a 2-L glass fermentor. Expression was induced by the addition of methanol and the enzyme was chromatographically purified using Sepharose FF and Q-source columns (GE Healthcare, Piscataway, NJ, USA) as reported elsewhere [34, 35].

### Kinetic studies

Kinetics of MMF, HMF, and DFF oxidation by AAO were studied by coupling the reaction of HRP and AmplexRed^®^ at 25 °C, in 100 mM sodium phosphate, pH 7.0. H_2_O_2_ released by AAO is used by HRP to oxidize AmplexRed^®^ to resorufin (Δε_563_ = 52,000 M^−1^ cm^−1^) in a 1:1 stoichiometric fashion. Therefore, spectrophotometric monitoring of the formation of colored resorufin allowed the indirect measurement of the AAO kinetic constants. Increasing concentrations of MMF (8–250 mM) were mixed with AAO (0.5 µM), AmplexRed^®^ (0.06 mM), and HRP (24 µg mL^−1^) at a final volume of 1 mL. Reactions were triggered by addition of AAO and followed in a Cary 4000 spectrophotometer (Agilent Technologies, Santa Clara, CA, USA). Kinetics were obtained from the linear phase of resorufin production as change in absorbance over time and averaged data for each substrate concentration were fitted to Michaelis–Menten equation to obtain the kinetic parameters using SigmaPlot software (Systat Software Inc., San Jose, CA, USA).

Residual activities of AAO and UPO were measured after different times of incubation in the presence of MMF and its oxidized derivatives. AAO residual activity was determined by following spectrophotometrically the production of *p*-anisaldehyde (Δε_285_ = 16,950 M^−1^ cm^−1^) [36] from 200 µM *p*-methoxybenzyl alcohol, in 1 mL of 50 mM sodium phosphate, pH 6.0, at 25 °C. Regarding UPO, its residual activity was measured as the veratraldehyde (Δε_310_ = 9300 M^−1^ cm^−1^) [32] produced from 10 mM veratryl alcohol and 2 mM H_2_O_2_, in 1 mL of 100 mM sodium phosphate, pH 7.0, at 25 °C. Experimentally determined values of residual activity were fitted to Eq. 1 describing the enzymatic activity loss as a function of time. This allowed estimation of the half-lives (Eq. 2) of AAO and UPO, as well as their TTN (Eq. 3, considering the enzyme half-life), TON (Eq. 4) and TOF (Eq. 5):

1 $${\text{res}} . {\text{ act}} . {\text{ = a}} \cdot {\text{e}}^{ - \lambda \cdot t}$$

2 $$t_{{\frac{1}{2}}} = \frac{\ln \;2}{\lambda }$$

3 $${\text{TTN = }}\frac{{k_{\text{cat}} \cdot t_{1/2} }}{\ln \;2}$$

4 $${\text{TON = }}\frac{\text{mol product}}{{{\text{mol}}\;{\text{catalyst}}}}$$

5 $${\text{TOF = }}\frac{\text{TON}}{\text{time}}.$$

### MMF oxidation reactions

MMF reactions were performed in 100 mM sodium phosphate (pH 7.0) under continuous shaking at 200 rpm in a thermostated chamber at 28 °C. In all of them, the substrate was added to a final concentration of 1.5 mM, while UPO and AAO final concentration was 5 µM. MOX attained a final concentration of 1 µM. All enzymes and substrates were added from the beginning of the reaction in the different reaction mixtures employed, except H_2_O_2_ (1.5 mM final concentration) and methanol (1 mM final concentration), which were gradually added after different incubation times (24, 48 and 72 h, and 72 and 96 h, respectively).

### GC–MS analyses

250-μL samples were harvested from the one-pot reactions after different times to analyze the products present. Reactions were stopped by adding HCl to give pH 2–3. Low pH values cause protonation of the organic acids and permit their liquid–liquid extraction, which was carried out by mixing the reaction mixtures with an excess of *t*-butyl-methyl-ether three times, followed by treatment with anhydrous NaSO_4_ to remove water traces. *t*-Butyl-methyl-ether was removed using a rotary evaporator at room temperature and samples were derivatized with 50 µL of BSTFA for 15 min at 25 °C [37].

**Fig. 5:**
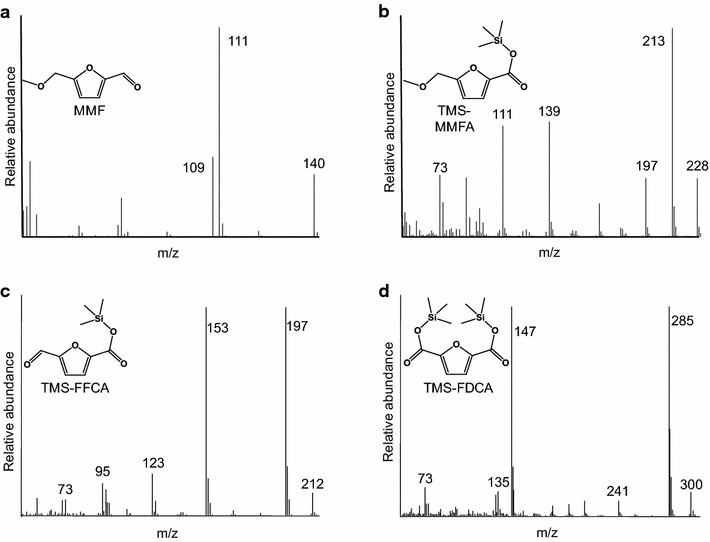
Mass spectra of authentic standards of the compounds involved in the cascade, as trimethylsilyl (TMS) derivatives. **a** MMF. **b** TMS-MMFA. **c** TMS-FFCA. **d** TMS-FDCA

Products were separated and identified using a gas chromatograph equipped with an HP-5MS column (Agilent, Santa Clara, CA, USA; 30 m × 0.25 mm internal diameter; 0.25 µm film thickness) coupled to a quadrupole mass detector. The oven program started at 110 °C (maintained for 2 min), increasing at 20 °C·min^−1^ until reaching 310 °C. Helium was used as the carrier gas at a flow rate of 1.2 mL min^−1^. The compounds involved in the MMF oxidative pathway were identified by comparing their mass spectra (and retention times) with those of derivatized authentic standards (Fig. 5). The following response factors were calculated as the slope of the fits of the responses of various concentrations of each standard compound (after its liquid–liquid extraction, derivatization and GC–MS analysis) to a linear equation: MMF: 1.7 × 10^7^ total-ion mM^−1^; MMFA: 4.1 × 10^7^ total-ion mM^−1^; FFCA: 3.1 × 10^7^ total-ion mM^−1^; and FDCA: 3.6 × 10^7^ total-ion mM^−1^. These response factors were used to estimate the mole percentage of each of the compounds in the reactions.

### NMR studies

^1^H-NMR was used to investigate the degree of hydration of the carbonyl group in the MMF molecule, using a Bruker 500 MHz instrument (Billenica, MA, USA). MMF (10 mM) was dissolved in 50 mM sodium phosphate (pD 7.0) prepared with ^2^H_2_O (99.9% isotopic purity). The internal reference for chemical shifts was the signal of the residual water proton (δ_H_ 4.9 ppm). Spectra of 10 mM MMF in DMSO-*d*6 (isotopic purity 99.98%) was run as a reference.

## Abbreviations

AAO: aryl-alcohol oxidase
BSTFA: N,O-bis(trimethylsilyl)trifluoroacetamide
DMSO-*d*6: deuterated dimethylsulfoxide
FDCA: 2,5-furandicarboxylic acid
FFCA: 5-formylfurancarboxylic acid
GC–MS: gas chromatography–mass spectrometry
HMF: 5-hydroxymethylfurfural
HMFCA: 5-hydroxymethylfurancarboxylic acid
HRP: horseradish peroxidase
*k*_cat_: catalytic constant
*k*_cat_/*K*_m_: catalytic efficiency
*K*_m_: Michaelis constant
MMF: 5-methoxymethylfurfural
MMFA: 5-methoxymethylfurancarboxylic acid
MOX: methanol oxidase
PEF: poly(ethylene-furandicarboxylate)
PET: poly(ethylene-terephthalate)
TOF: turnover frequency
TON: turnover number
TTN: total turnover number
UPO: unspecific peroxygenase
